# Neural circuits regulating activity-based anorexia

**DOI:** 10.1038/s41386-026-02403-4

**Published:** 2026-04-07

**Authors:** Dongmin Yoon, Stephanie Dulawa

**Affiliations:** Department of Psychiatry, University of California San Diego, La Jolla, CA USA

**Keywords:** Diseases of the nervous system, Behavioural methods, Translational research

## Abstract

Evolutionarily conserved neural circuits evolved that mediate switching between feeding and foraging for food, depending on environmental conditions such as food scarcity and internal state [1–3]. Activity-based anorexia is a phenomenon observed ubiquitously in normal mammals that emerges under conditions of time-restricted food availability and continuous access to running wheels. Under these experimental conditions, rodents progressively lose body weight and develop paradoxical hypophagia and compulsive wheel running, which can prove fatal if left unchecked. On the other hand, rodents survive indefinitely under conditions of either time-restricted food access or running wheel availability. In this review, we discuss preclinical studies within the past decade which used modern genetic circuit-dissecting tools including chemogenetic, optogenetic, and calcium imaging, to dissect the neural circuitry modulating activity-based anorexia. We highlight how circuits interconnecting the hypothalamus, prefrontal cortex, amygdala, mesolimbic system, and monoaminergic nuclei, interact to modulate animals’ decision to feed or forage in the activity-based anorexia paradigm. We then highlight how these recent findings have aided in identifying pathophysiological mechanisms underlying neuropsychiatric disorders characterized by the maladaptive prioritization of exercise over feeding. Finally, we suggest approaches for the development of targeted therapeutics for anorexia nervosa, which has no approved pharmacological treatments.

## Introduction

Neural mechanisms evolved that allow animals to adaptively switch between feeding and foraging for food, depending on environmental conditions and internal states. Essential for survival, these neural mechanisms permit the prioritization of either feeding or foraging during times of food abundance or scarcity, positive or negative energy balance, and safe versus risky environments [4]. In the activity-based anorexia (**ABA**) paradigm, rodents are singly housed and exposed to both time-scheduled food restriction and constant running wheel access, which results in progressive hyperactivity, hypophagia, and weight loss [5, 6]. Yet, rodents exposed only to time-scheduled food restriction, or constant running wheel access, maintain body weight indefinitely [5, 6]. Thus, rodents exposed to ABA conditions voluntarily restrict food intake compared to controls exposed to scheduled feeding without running wheel access; in addition, rodents exposed to ABA conditions run more than controls fed ad lib with wheel access [5–10]. If ABA conditions remain unchecked, rodents will develop hypothermia, increased HPA axis activity, and ultimately stomach ulceration and death [5, 11]. Exposure to the ABA paradigm also produces changes in cognitive states, such as increased cognitive rigidity [12]. Many animal species including mice, rats, hamsters, gerbils, guinea pigs, and chipmunks develop ABA under these conditions [6, 13, 14], suggesting that ABA behavior may be adaptive in certain environments, such as during food scarcity to facilitate migration [15]. A heightened propensity to develop ABA behavior during low energy states has been theorized to underlie aspects of some neuropsychiatric disorders, especially anorexia nervosa (**AN**) [16, 17]. Similar to ABA behavior, core behavioral features of AN are the restriction of food intake and compulsive exercise that persist despite extreme and even life-threatening weight loss. Of all neuropsychiatric disorders, the ABA phenomenon may have most relevance to AN; however, the ABA phenomenon might also have relevance to aspects of additional neuropsychiatric disorders, including avoidant/restrictive eating disorder (ARFID), and obsessive compulsive disorder (OCD) [18–20].

Until recently, studies investigating the neural mechanisms underlying ABA used traditional anatomical, pharmacological, or genetic techniques, such as systemic drug treatments and constitutive genetic manipulations [21–26]. The past decade has seen a dramatic rise in the use of modern neural circuit-dissection techniques to study behaviors of interest. For example, in vivo fiber photometry has been used to measure the bulk activity of specific neuronal populations, while both chemogenetic and optogenetic techniques have been used to manipulate the activity of specific neuronal populations. These approaches are rapidly advancing our understanding of the neural circuits that regulate ABA.

## Neural circuitry of activity-based anorexia

Here, we review work using modern genetic circuit-dissection techniques to elucidate the neural circuitry of ABA, which consists of approximately ten reports over the past decade. These studies have examined the role of hypothalamic agouti-related peptide (**AgRP**)- or proopiomelanocortin (**POMC**)-expressing neurons [27, 28], lateral hypothalamic (LH) neurons [29], nucleus accumbens (**NAc**) medium-spiny neurons [30, 31], central extended amygdala (**EAc**) neurons [32], ventral tegmental area (**VTA**) projections to the NAc [33] or dorsal raphe [34], and medial prefrontal cortical (**mPFC**) neuron projections to the NAc [12] or dorsal raphe [35]. We also discuss contributions of this preclinical work to identifying mechanisms underlying neuropsychicatric disorders including AN, approaches for developing targeted therapeutic interventions, and future research directions.

### Hypothalamic cell groups

Hypothalamic AgRP-expressing and POMC-expressing neurons reciprocally regulate feeding and metabolism [36, 37], and both were recently probed for their roles in regulating ABA. Recent work by and Miletta et al. [28] and Sutton et al. [27] examined the role of AgRP neurons in modulating ABA. Photometry studies have shown that food deprivation progressively increases the activity of AgRP neurons [38, 39], while the onset of feeding, or even the sensory detection of food, robustly reduces activity of these neurons [40, 41]. Sutton et al. [27] used photometry to measure calcium activity in AgRP neurons of mice behaving in the ABA paradigm. During time-scheduled food access, mice could obtain food pellets by nose-poking into a feeding experimentation device 3 (FED3) device placed in the cage. Calcium signals collected 7.5–10 s after pellet retrieval during the first 30 min of food access were averaged and compared between three groups: mice with wheel access and time-scheduled feeding (ABA), mice with wheel access and ad lib feeding (RUN), and mice with time-scheduled feeding but no wheels (STARV). On day 1 of the ABA paradigm, the normal reduction in AgRP neuronal activity normally observed just following pellet retrieval was diminished in both ABA and RUN groups compared to the STARV group. Interestingly, on day 4 of ABA, the ABA group regained the normal attenuation of AgRP neuronal activity following pellet retrieval, and only the RUN group showed loss of the normal reduction in AgRP neural activity. Normalization of the AgRP neural response to food in ABA mice might have resulted from ongoing weight loss. Next, chemogenetic inhibition of AgRP neurons was performed in mice expressing hM4Di on AgRP neurons, via daily CNO injections given1 hour before food delivery. STARV and ABA groups showed increased weight loss while RUN mice did not, however no changes in food intake was observed in any group. Additionally, only ABA mice showed increased wheel running. Finally, AgRP neurons were chemogenetically activated using systemic CNO injections in mice expressing hM3Dq on AgRP neurons, to either prevent or reverse ABA development. When CNO treatment and ABA conditions were initiated simultaneously, AgRP neural excitation increased food intake and body weight, but had no effects on wheel running in all groups. In a separate study assessing mice only in the ABA condition, CNO treatment was initiated in half of the mice after 4 days of ABA, when mice had already lost weight. Chemogenetic activation of AgRP neurons reversed ABA progression by decreasing total and dark cycle running and increasing food intake and meal size. Thus, chemogenetic activation of AgRP neurons both attenuates and reverses ABA by promoting food-seeking behaviors through integration of peripheral metabolic signals.

Work by Miletta et al. [28] is partially consistent with these findings [27], with some caveats. They found that neonatal AgRP neuronal ablation reduced bodyweight during ABA in adulthood, similar to chemogenetic AgRP neuronal inhibition. However, mice with neonatal AgRP ablation also showed robust reductions in both food intake and wheel running during ABA. Ablated mice also lacked normal fuel mobilization during ABA; and when given access to a high-fat diet, their dramatic weight loss during ABA conditions was prevented. However, other reports have shown that even intact rodents show robust reductions in ABA behavior when high-fat diet is provided [42], indicating that highly palatable foods prevent ABA overall. Miletta et al. [28] also found that activation of AgRP neurons using the transient receptor potential cation channel subfamily V member 1 (Trpv1) paired with capsaicin injections, improved and running endurance during ABA, but did not alter survival or food intake. Finally, Miletta et al. [28] also showed using fiber photometry that voluntary cessation of a running bout by mice coincides with a reduction in AgRP neuronal activity.

Daimon and Hentges (2022) have performed the only investigation into the role of POMC neurons in ABA. Chemogenetic inhibition of POMC neurons selectively decreased food anticipatory activity (FAA), defined as wheel running during the 4 h period preceding food delivery. However, POMC inhibition had no effect on food intake, body weight, or total wheel running during ABA paradigm [43, 44].

Schele and Stoltenborg et al. [29] combined Fos-targeted recombination in active populations 2 (Fos-TRAP2) and chemogenetic techniques to establish that a neural ensemble spanning the length of the LH becomes activated during ABA, and promotes feeding, running, and weight gain in female mice [29]. In an elegant design, TRAP2 mice (Fos2A-iCreER transgenic mice) received infusions of a cre-dependent AAV expressing either hM4Di, hM3D(Gq), or mCherry into either the LH or lateral septum (LS). Injection of these mice with 4-hydroxytamoxifen (4-OHT) triggers the permanent Cre-dependent expression of DREADDs only in activated neurons. Mice were first exposed to either ABA, or wheel only conditions, and upon reaching a weight-loss criteria of approximately 25%, 4-OHT was administered to “TRAP” or tag activated neurons. Next, ad lib feeding was re-established, and mice received injections with CNO to activate DREADDs, thereby either reactivating or inhibiting neurons which were tagged during ABA or control conditions. Results showed that reactivation of tagged LH neurons induced robust increases in feeding and running, and resulted in an increase in bodyweight relative to controls. On the other hand, hM4Di-mediated inhibition of tagged neurons in the LH reduced both food intake and running, but did not alter bodyweight. Neither activation nor inhibition of tagged LS neurons altered measures of ABA. These findings suggest that neurons activated by ABA in the LH drive food seeking and food intake to increase bodyweight and promote survival. Interestingly, previous work indicates that the anticipation of meals during food restriction, which is linked to ghrelin signaling [22], more strongly affects activation in the hypothalamus than negative energy balance alone [45].

### Central extended amygdala protein kinase C delta-expressing neurons

Previous work has shown that neurons expressing protein kinase C-delta (PKC-δ) in two distinct nuclei within the EAc: the central nucleus of the amygdala (**CeA**) and the oval region of the bed nucleus of the stria terminalis (**ovBNST**), regulate many anorexigenic signals and reduce feeding upon their activation [46–48]. Recently, Schnapp et al. [32] identified an essential role for PKC-δ expressing neurons within the CeA and ovBNST in the development of ABA, using genetic ablation and fiber photometry techniques in behaving mice.

Schnapp et al. [32] ablated PKC-δ expressing neurons using local infusion of a Cre-dependent adeno-associated virus (**AAV**) expressing taCasp3 into PKC-δ Cre mice. Virus was infused into both the CeA and ovBNST, or into either site alone. Under control conditions, neuronal ablation had no effect on body weight, food intake, wheel running, or measures of energy balance. However, only ablation of PKC-δ expressing neurons within both the CeA and ovBNST prevented the development of ABA. Dual ablation prevented bodyweight loss, reductions in food intake, and increases in running under ABA conditions. Additionally, ablation of PKC-δ expressing neurons only within the CeA also improved survival.

Next, Schnapp et al. [32] recorded calcium activity using fiber photometry from PKC-δ expressing neurons in the CeA or the ovBNST of normal mice during ABA. Using a within subject design, calcium activity was recorded from each mouse during ad lib feeding with wheel access, on day 1 of ABA, and once mice lost 20% of their initial body weight. Calcium activity was robustly increased in PKC-δ expressing neurons of both nuclei during ABA, compared to during only wheel access [27]. For these studies, Schnapp et al. [32] reported averaged calcuim fluorescence over 30 min beginning at food delivery, while most other studies analyzed averaged calcium activity over seconds, immediately following food retreival [27]. Thus, the increase in calcium activity reported here reflects pellet delivery, pellet retrieval, and pellet consumption, as well as other behaviors performed within the first 30 min of daily food availability.

### Ventral tegmental area projections to the NAc or dorsal raphe

The VTA to NAc projection modulates feeding, movement, and reinforcement [49–55]. Foldi et al. [33] used a dual virus approach to activate this pathway; a Cre-dependent AAV expressing hM3Dq was infused into the VTA, and a retrograde canine adenovirus expressing Cre was infused into the NAc for retrograde transport to VTA cell bodies. Ten days later, rats were assessed for the effects of VTA→NAc pathway activation on ABA development, and recovery from ABA. To assess effects on ABA development, rats received one daily systemic CNO injection, 30 min before food presentation. In a separate study, rats were subjected to ABA conditions and after losing 14.5% of their baseline body weight, CNO was administered daily. Chemogenetic activation of the VTA→NAc pathway prevented the development of ABA, increased food intake and FAA, but did not alter wheel running. Furthermore, initiating chemogenetic activation of this pathway after the development of ABA also increased survival by prevented further weight loss, while food intake and wheel running were not altered. This study by Foldi et al. [33] established a role for the VTA→NAc projection in the regulation of ABA; however, more work is required to determine the role of different neuronal subtypes in this effect, including those using dopamine, GABA, and glutamate as neurotransmitters, and those using more than one neurotransmitter.

Both dopaminergic and serotonergic neurons regulate feeding and motivated behavior [56–60], and how these neuronal types interact to modulate ABA behavior was recently investigated by Cai et al. [34] using photometry and chemogenetics. They reported that during ABA, dorsal raphe nucleus (**DRN**)-projecting dopaminergic VTA (**DA**^**VTA**^) neurons showed highly elevated calcium signals both during fasting and refeeding. Next, by injecting a cre-dependent hM4Di-expressing retrograde AAV into the DRN of DAT-CreER mice, they examined whether chemogenetic inhibition of DRN-projecting DA^VTA^ neurons would reduce the ABA phenotype. Compared to mice receiving control injections, mice receiving CNO showed increased survival. Although food intake was increased and wheel running was decreased, only averaged values for the entire ABA period were used for comparison. Since survival time varied considerably between animals, this approach of comparing overall means might have obscured results.

Next, Cai et al. [34] found that ABA conditions increased calcium signals in serotonergic neurons of the DRN (**5-HT**^**DRN**^), even during food delivery, and chemogeneic inhibition of 5-HT^DRN^ neurons improved survival during ABA. Cai et al. [34] then dissected the role of dopamine D1 (**DRD1**) versus D2 (**DRD2**) receptors expressed on 5-HT^DRN^ neurons in modulating ABA behavior. Compared to control mice, mice lacking DRD1 only in 5-HT^DRN^ neurons showed reduced activity of these neurons and increased survival and during ABA, while mice lacking DRD2 only in 5-HT^DRN^ neurons did not show changes in behavior during ABA [34]. Consistent with findings in mice lacking DRD1 on 5-HT^DRN^ neurons, daily systemic injection of normal mice with the DRD1 antagonist SCH23390 (0.1 mg/kg) 30 min before food delivery also increased survival during ABA [34]. However, a previous report by Klenotich et al. (2015) did not find any effect of 0.005–0.5 mg/kg/day SCH23390 on ABA; although, drug was administered continuously in the drinking water, and might not have achieved peak levels sufficient to produce effects induced by acute injection [21]. In sum, reducing activity at DRD1 on 5-HT^DRN^ neurons, or chemogenetic inhibition of 5-HT^DRN^ neurons, reduces ABA.

### Nucleus accumbens medium spiny neurons

The vast majority of neurons in the NAc are GABAergic medium spiny cells which express dopamine D1 and/or D2 receptors, and receive inputs from many brain regions including the VTA. Walle et al. (2024) employed an innovative strategy using a bitransgenic line expressing the flippase recombinase in D1-exressing neurons, and Cre recombinase in D2-expressing neurons. Thus, intra-NAc core infusions of a 1:1 mixture of Cre-dependent and flp-dependent viruses, each expressing *either* hM3Dq *or* hM4Di depending on the experiment, allowed CNO treatment to induce simultaneous activation of D1-MSNs and inhibition of D2-MSNs, or the opposite. In one experiment, CNO was injected systemically 30 min before food delivery during ABA, resulting in simultaneous increases in D1-MSN and decreases in D2-MSN neuronal activity [30]. This manipulation increased weight loss, and robustly increased wheel running compared to controls. However, food intake or survival was not altered. When the opposite manipulation was performed by simultaneously decreasing D1-MSN and increasing D2-MSN neuronal activity, CNO treatment reduced weight loss and wheel running, without altering food intake or survival. However, mice with simultaneous D1-MSN activation and D2-MSN inhibition did show reduced survival compared to mice with simultaneous D1-MSN inhibition and D2-MSN activation [30]. Thus, shifting the balance of activity towards D1-MSNs and away from D2-MSNs within the NAc core drives ABA behavior. This work was performed using male mice, and requires extension into females.

Another study compared the effects of D2 receptor overexpression in the NAc core on ABA in both sexes. Welch et al. (2019) virally overexpressed D2 long (**D2L**) postsynaptic receptors on D2-MSNs of the NAc core, and found that survival in the ABA paradigm was dramatically reduced in female, but not male, mice [31]. Although the activity of D2-MSNs were not directly assessed in this study, D2 receptor overexpression might inhibit neuronal activity, since D2 receptor are inhibitory to neurons when activated. Consistent with these findings, AN patients also show increased dopamine D2/D3 receptor binding in the anteroventral striatum as measured by [11 C] raclopride binding using positron emission tomography [61]. These findings suggest that the manipulations to D1- versus D2-MSNs performed by Walle at al. (2024) might yield robust sex differences if performed in both sexes. In sum, the interplay of activity of D1- versus D2-MSNs strongly modulates the expression of ABA.

### Medial prefrontal cortical projections to the NAc or dorsal raphe

The medial PFC sends dense projections to brain regions exerting a powerful influence on the expression of ABA, including the NAc and dorsal raphe [62–65]. Two recent studies examined a role for mPFC projections in modulating ABA in mice. Milton et al. (2020) infused a retrograde AAV expressing Cre recombinase into the AcbSh, and a Cre-dependent AAV expressing either the excitatory designer receptor exclusively activated by designer drugs (**DREADD**) hM3D(Gq), or the inhibitory DREADD hM4Di, or only a fluorophore into the mPFC. Clozapine-N-oxide (**CNO**) was administered 30 min before food delivery during the ABAparadigm to increase, decrease, or not alter the activity of the mPFC→AcbSh projection. Chemogenetic inhibition of the mPFC→AcbSh projection robustly increased survival and prevented weight loss, while chemogenetic activation of the pathway increased running, and nonsignificantly accelerated drop out from the paradigm. Neither chemogenetic manipulation altered food intake. When food remained available *ad lib*, no effects of either chemogenetic manipulation was observed. Thus, Milton et al. (2020) found that chemogenetic inhibition of the mPFC→AcbSh projection protects against ABA.

Du et al. (2022) explored the role of mPFC pyramidal neurons projecting to the dorsal raphe in ABA modulation, following the induction of ABA behavior in adolescent mice. Mice received infusions of a Cre-dependent hM3DGq AAV into the mPFC, and a retrograde AAV expressing Cre recombinase into the dorsal raphe. The DREADD agonist 21 was injected systemically twice daily, and approximately 44% of mPFC to dorsal raphe neurons were activated, but ABA measures including bodyweight were not affected. Investigators also found increased Fos expression in GABAergic interneurons in the mPFC of chemogenetically activated mice, suggesting that activation of the pathway of interest was suboptimal. A role for the mPFC→dorsal raphe projection in ABA remains to be established.

## Activity-based anorexia in neuropsychiatric disorders

The ABA phenomenon has been used to investigate certain core features of AN, including restriction of food intake and compulsive exercise despite ongoing weight loss. ABA also recapitulates additional features of AN, including increased vulnerability with younger age [66] and decreased cognitive flexibility [12, 67, 68]. However, ABA does not mimic many other aspects of AN such as distorted body image, and ritualistic behaviors around food. Studying ABA circuitry might also reveal mechanisms of other neuropsychiatric disorders. For example, ARFID, which is a new diagnosis introduced in DSM-V, is also characterized by hypophagia, low body weight, and sometimes, overexercise [69]; some evidence suggests that ARFID can progress into AN [70]. Both disorders are highly comorbid with OCD, and AN has the highest SNP-based genetic correlation with OCD compared to any other neuropsychiatric disorder [71, 72]. Interestingly, non-depressed OCD patients show significantly lower body weight than healthy controls [73–76].

## Anorexia nervosa

AN heritability is estimated to be approximately 70%, although the genetic basis of AN remains to be elucidated [77]. The largest genome-wide association study (**GWAS**) of AN to date identified eight risk loci [71]; yet, the genetic variants responsible for these signals remain to be confirmed. The genetic architecture of AN shows significant genetic correlations with psychiatric disorders (especially OCD), and also with physical activity, metabolic, and anthropometric traits, independent of the effects of common variants associated with body-mass index. Once the risk genes underlying these GWAS signals are identified, mouse genetic models can be generated to examine their influence on ABA circuitry and behavior.

These findings from preclinical circuit-dissecting studies of ABA have identified potential mechanisms underlying the alterations to homeostatic and hedonic processes in AN. Homeostatic processes regulated by the ventricular hypothalamus normally increase hunger during negative energy balance and modulate basic metabolic processes to permit survival, while hedonic processes drive reward-based or pleasure-seeking behaviors such as consuming palatable food regardless of energy needs [78, 79]. Both processes are likely altered in AN, as starvation and underweight stimuli become rewarding, and homeostatic hunger signals are overridden [80–82]. Further, extreme exercise becomes highly rewarding despite severe weight loss [83, 84]; although, other evidence suggests that excessive exercise might not reflect a reward-driven behavioral addiction, but could rather reflect a compulsive behavior which serves to reduce the anxiety and negative affect characteristic of AN [85, 86].

The report that the normal increase in AgRP neuron firing during food deprivation is attenuated during ABA, and that chemogenetic restoration of AgRP neural activity can prevent or reverse ABA behavior, reveals a potential mechanism that could underlie reduced homeostatic feeding in AN [27]. Additionally, disruptions within a ventral limbic circuit including the amygdala, anterior insula, and anterior ventral striatum/NAc that normally drives reward-seeking behaviors has also been implicated in AN [81, 87]. The finding that stressor-sensitive PKC-δ expressing neurons within the CeA and ovBNST are robustly activated by ABA conditions, and that their ablation attenuates hypophagia and wheel running in the ABA paradigm, is consistent with evidence that compulsive exercise may serve to reduce anxiety and negative affect in AN [85, 88]. Likewise, the observation that serotonergic neurons of the dorsal raphe have increased activity during ABA, and that chemogenetic inhibition of these neurons reduces ABA, is in line with evidence for a link between anxiety and serotonergic overactivity in AN. In particular, increased activity of dorsal raphe serotonergic neurons can promote anxiety-like behavior [89], and long-term weight-restored AN patients exhibit elevated concentrations of cerebrospinal fluid 5-hydroxyindoleacetic acid (5-HIAA), the primary serotonin metabolite, suggesting increased serotonin activity [90, 91].

The prominent theory that hypoactive reward systems underlie hypophagia in AN is supported by the finding that chemogenetic activation of the mesolimbic VTA→NAc pathway prevents the development of ABA through increased food intake [33]. Interestingly, the chemogenetic reduction in D2-MSN activity and increase in D1-MSN activity within the NAc core dramatically increased reward from wheel running [30], similar to overexercise AN. Intriguingly, overexpression of D2 receptors on NAcC D2-MSNs appeared to shift motivation away from feeding and towards wheel running, rapidly reducing survival during ABA, but only in female mice [31]. Similarly, female AN patients show increased dopamine D2/D3 receptor binding in the anteroventral striatum as measured by [11 C] raclopride binding using positron emission tomography [61]. These findings in humans and mice suggest that D2-MSNs, or “the indirect pathway” within the NAc, may be more inhibited in AN. Indeed, inhibition of NAc D2-MSNs also results in impaired reversal learning [92] and reduced aversive learning [93], phenotypes which are consistent with the cognitive rigidity and insensitivity to losses in AN patients [80, 94]. Lastly, a dorsal executive function neural circuit including the dorsolateral prefrontal cortex (DLPFC) in humans and the mPFC in rodents that modulates selective attention, planning, and effortful affective regulation is thought to be overactive in AN, resulting in a strategic rather than hedonic approach to responding to reward stimuli [80, 95]. The finding that chemogenetic inhibition of the “top-down” mPFC→AcbSh projection reduces ABA supports this notion. Interestingly, this same chemogenetic manipulation also increased cognitive flexibility in a reversal learning task [67]. Recently, a novel viral-based translating ribosome affinity purification technique was used to identify transcriptional differences within the mPFC→AcbSh pathway between rats that were more or less susceptible to weight loss in the ABA paradigm, potentially identifying novel targets for treatment development [96].

## Therapeutic directions

Current treatment guidelines for AN recommend family-based treatments as a primary treatment [97–99], while the efficacy of other psychosocial therapies are under investigation [100–102]. At present, outcomes in AN remain unacceptably poor [103]. No approved pharmacological treatments for AN exist [104]. Although pharmaceutical agents are often prescribed off label to treat comorbid conditions such as depression and anxiety in hopes of indirectly improving AN symptoms [103], no class of agent including antidepressants [105], antipsychotics [106], mood stabilizers [107], or benzodiazepines [108] consistently improve weight gain in underweight AN patients in randomized placebo-controlled randomized studies.

Some agents targeting neurotransmitter systems that modulate ABA are under investigation as therapies for AN. For instance, the psychedelic psilocybin is a direct agonist at serotonin 2 A and 1 A receptors, and increases cognitive flexibility and reduces weight loss in rodents in the ABA paradigm [109]. A recent open-label clinical trial examined effects of psilocybin in 10 subjects with AN [110]. While AN patients reported increased quality of life and significant improvements in Eating Disorder Examination subscale, no significant improvements in body mass index were found. Although antipsychotics do not improve weight gain in AN, these agents vary in their neurotransmitter receptor targets [111, 112] and thus vary in their effects on AN. The partial dopamine D2 receptor agonist aripiprazole has shown some promise in improving weight gain in AN, as revealed by retrospective chart reviews and case series studies [113–116]. Additionally, several studies have reported that low doses of ketamine reduce ABA in rodents [117, 118]; however, no randomized controlled trials for ketamine in AN have been reported. Randomized placebo controlled trials assessing effects of the above agents on weight gain in AN are sorely needed.

Recently, interest in non-invasive brain stimulation as a novel treatment for AN has grown. Brain stimulation approaches for relatively shallow cortical regions include repetitive transcranial magnetic stimulation (**rTMS**) and transcranial direct current stimulation. When these methods are ineffective, deep brain stimulation (**DBS**) can provide effective treatment. A recent meta-analysis reported that rTMS targeting the dorsolateral prefrontal cortex, dorsomedial prefrontal cortex, insula, or the inferior parietal lobe revealed a significant increase in body mass index (**BMI**) and reduction of core symptoms of AN [119]. A meta-analysis of eleven DBS studies in AN found that stimulation of the subcallosal cingulate cortex, as opposed to the ventral anterior limb of the internal capsule or the nucleus accumbens, was the most supported stimulation location based on BMI improvement 6 -12 months post-treatment [120]. These encouraging early findings in the treatment of AN using brain stimulation warrant further studies, and could be complimented by preclinical studies since these neurostimulation approaches are also possible in rodents [121–125].

## Future research directions

**Fig. 1:**
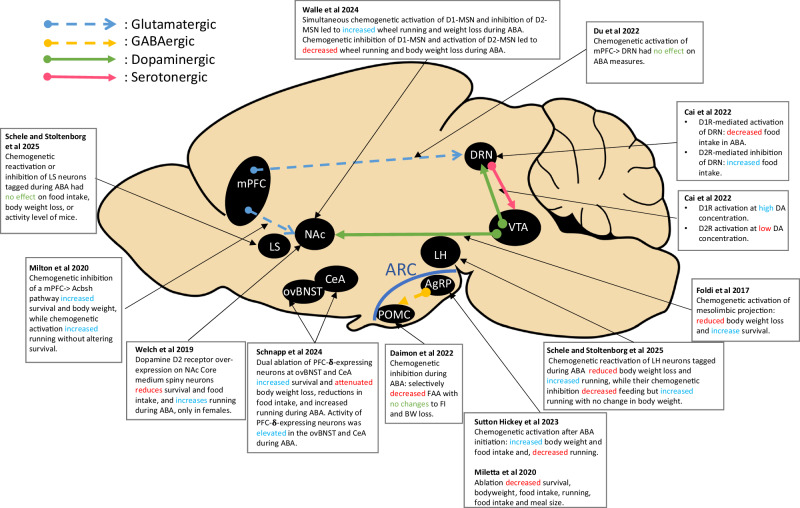
Brain regions and neural projections that modulate activity-based anorexia. Blue: Glutamatergic excitatory projection. Yellow: GABAergic inhibitory projection. Green: Dopaminergic projection. Pink: Serotonergic projection. mPFC medial prefrontal cortex, DRN dorsal raphe nucleus, VTA ventral tegmental area, NAc nucleus accumbens, CeA central amygdala, ovBNST oval region of bed nucleus of the stria terminalis, POMC proopiomelanocortin, AgRP agouti-related peptide, Acbsh nucleus accumbens shell, ARC arcuate nucleus of the hypothalamus, LH lateral hypothalamus, LS lateral septum, FAA food anticipatory activity.

Since adaptively switching between feeding and foraging for food is essential for survival, it is unsurprising that numerous neuromodulators, neurotransmitters, and distinct brain regions and circuits modulate these behaviors (Fig. 1); however, it is surprising that the balance between feeding and physical activity can become fatally disrupted during ABA conditions and in neuropsychiatric conditions such as AN. Several conceptual or methodological future directions could be taken to improve our understanding of the neural circuits that modulate ABA.

Experiments recording bulk calcium activity from neurons modulating feeding and/or wheel running have measured neural activity primarily during food delivery in the ABA paradigm, but rarely during wheel running initiation or cessation. Measuring neural activity during other behaviors aside from feeding will be essential to understand the ABA phenomenon. Furthermore, developing innovative variants of the ABA paradigm itself could yield important information. For example, the precise time at which rodents decide to feed or forge is unclear in the current experimental set up. If the paradigm was modified so the animal was required to nose poke to gain access to food and the wheel in separate chambers, neural activity could be measured during the nose poke or “decision period”. While neural activity has primarily been measured in bulk using fiber photometry during ABA, head-mounted miniscopes could be used to record from groups of neurons. Chemogenetics has primarily been used to alter neural activity during the ABA, largely due to not requiring any attachments to the animal; however, this approach does not permit precisely timed manipulation of neural activity. Application of wireless optogentics, and/or closed-loop optogenetic approaches could shed light on the mechanisms underlying the decision to feed or forge. Although feeding experimentation device 3 (**FED3**) devices have recently been used to collect fine-grained food intake data synchronized with photometry recording [27], it should be noted that requiring operant performance to acquire each food pellet introduces other confounds into natural foraging behavior. Thus, experiments assessing the neural activity during free feeding will also be essential. Lastly, more consensus on statistical approaches for analyzing ABA data would be helpful for the field. During ABA, animals are removed from the paradigm when they reach weight loss criteria, such that the number of animals per group for each measure decreases progressively over time. Some studies have reported at least 2 weeks of ABA data, which can be analyzed using general linear mixed models to handle missing data [21, 23, 31]. However, other studies have truncated ABA data to as little as 3 days to analyze data using standard methods. In sum, a more nuanced understanding of the neural circuits underlying ABA could provide foundational knowledge regarding the mechanisms underlying aspects of neuropsychiatric disorders including AN, and lead to effective treatments.
